# Supplementary material for: Elevated prevalence of Helicobacter species and virulence factors in opisthorchiasis and associated hepatobiliary disease
Source: Sci Rep. 2017 Feb 15;7:42744. doi: 10.1038/srep42744 (PMC5309894; doi:10.1038/srep42744)
Supplement: Supplementary Information [file srep42744-s1.pdf]

Elevated prevalence of *Helicobacter* species and virulence factors in opisthorchiasis and associated hepatobiliary disease

Raksawan Deenonpoe[1,2], Eimorn Mairiang[3], Pisaln Mairiang[4], Chawalit Pairojkul[2], Yaovalux Chamgramol[2], Gabriel Rinaldi [5,♯], Alex Loukas[6], Paul J. Brindley[5], Banchob Sripa[1,2]

[1] Tropical Disease Research Laboratory, Faculty of Medicine, Khon Kaen University, Khon Kaen 40002, Thailand

Departments of [2]Pathology, [3]Radiology and [4]Medicine, Faculty of Medicine, Khon Kaen University, Khon Kaen, 40002, Thailand

[5] Department of Microbiology, Immunology and Tropical Medicine, and Research Center for Neglected Tropical Diseases of Poverty, School of Medicine & Health Sciences, The George Washington University, Washington DC, 20037, USA

[6] Centre for Biodiscovery and Molecular Development of Therapeutics, Australian Institute of Tropical Health & Medicine, James Cook University, Cairns, Queensland 4878, Australia

♯ Current address: Sanger Institute, Wellcome Trust Genome Campus, Cambridge CB10 1SA, United Kingdom

Correspondence: Banchob Sripa, Tropical Disease Research Laboratory, Department of Pathology, Faculty of Medicine, Khon Kaen University, Khon Kaen 40002, Thailand, email banchob@kku.ac.th; or Paul J. Brindley, Department of Microbiology, Immunology and Tropical Medicine, and Research Center for Neglected Tropical Diseases of Poverty, School of Medicine & Health Sciences, The George Washington University, Washington DC, 20037, USA, email, pbrindley@gwu.edu

Recent reports suggest that *Opisthorchis viverrini* serves as a reservoir of *Helicobacter* and implicate *Helicobacter* in pathogenesis of opisthorchiasis-associated cholangiocarcinoma (CCA). Here, 553 age-sex matched cases and controls, 293 and 260 positive and negative for liver fluke *O. viverrini* eggs, of residents in Northeastern Thailand were investigated for associations among infection with liver fluke, *Helicobacter* and hepatobiliary fibrosis. The prevalence of *H. pylori* infection was higher in *O. viverrini*-infected than uninfected participants. *H. pylori* bacterial load correlated positively with intensity of *O. viverrini* infection, and participants with opisthorchiasis exhibited higher frequency of virulent *cag*A-positive *H. pylori* than those free of fluke infection. Genotyping of *cagA* from feces of both infected and uninfected participants revealed that the AB genotype accounted for 78 % and Western type 22 %. Participants infected with *O. viverrini* exhibited higher prevalence of typical Western type (EPIYA ABC) and variant AB'C type (EPIYT B) CagA. Multivariate analyses among *H. pylori* virulence genes and severity of hepatobiliary disease revealed positive correlations between biliary periductal fibrosis during opisthorchiasis and CagA and CagA with CagA multimerization (CM) sequence-positive *H. pylori*. These findings support the hypothesis that *H. pylori* contributes to the pathogenesis of chronic opisthorchiasis and specifically to opisthorchiasis-associated CCA.

Introduction

Infection with the fish-borne liver fluke *Opisthorchis viverrini* is endemic in Southeast Asia including regions of the Lao People's Democratic Republic, Thailand, Cambodia and Vietnam [1-3]. Opisthorchiasis is associated with hepatobiliary morbidity including chronic cholangitis, cholelithiasis, periductal fibrosis and bile duct cancer, or cholangiocarcinoma (CCA) [4-7]. Khon Kaen province in Northeast Thailand has reported the highest incidence of CCA in the world, greater than 100 cases per 100,000 residents [8]. Chronic inflammation in response to metabolites and growth factors released by this parasitic worm and related phenomena are implicated in the pathogenesis of liver fluke infection-associated hepatobiliary diseases [7,9-12]. However, the biliary morbidity in the setting of opisthorchiasis may not be solely linked with liver fluke infection; other factors including carriage of *Helicobacter* and other microbiome changes within the biliary tract might participate [13].

More than 30 species of *Helicobacter* have been described [14] and *H. pylori* was the first bacterial pathogen confirmed to cause gastric disease including peptic ulcer, gastric lymphoma and gastric adenocarcinoma [15-19]. On the other hand, carriage of *H. pylori* occurs in at least half the human population with transmission from mother to child and other routes. Indeed the human-*H. pylori* association likely is at least 100,000 years old [20], an association that appears to be beneficial in early life, including contributions to a healthy microbiome and reduced early-onset asthma [21,22]. Infection with species of *Helicobacter* has been implicated in other malignant and benign diseases of the biliary tract [23-28]. Virulence factors of *H. pylori* including cytotoxin-associated gene A (*cag*A), *cag*E and vacuolating cytotoxin A (*vac*A) participate in the pathogenesis of these conditions [29]. The related species *H. hepaticus* and *H. bilis* also associate with hepatobiliary diseases [30-32].

Opisthorchiasis may enhance colonization of the biliary tree by species of *Helicobacter* in like fashion to other changes in the biliary microbiome [33]. The influence of opisthorchiasis on cholestasis as a consequence of the liver fluke migration and establishment within the bile ducts provide explanations for bacterial colonization leading to bacterial cholangitis [34]. In addition, the migration of the flukes themselves from the external environmental through the alimentary tract and into the biliary tract might convey bacterial passengers, both on the external surface of the trematode and within the gut of the parasite [35-38].

We recently reported, in a hamster model of liver fluke infection-induced biliary disease, higher prevalence and intensity of co-infection with *H. pylori* and *H. bilis* in *O. viverrini*-infected compared to uninfected hamsters, suggesting that this liver fluke serves as a reservoir for *H. pylori*[37]. Here we undertook a human study with more than 500 residents in villages of four provinces of northeastern Thailand endemic for opisthorchiasis[3]. Liver fluke infection was associated with a higher frequency of *cagA*-positive *H. pylori*. Moreover, the presence of *H. pylori cag*A gene as well as alleles of was associated with increased morbidity, specifically periductal fibrosis of the biliary tree. These findings support the hypothesis that *H. pylori* contributes to the pathogenesis of chronic opisthorchiasis and specifically to opisthorchiasis-associated cholangiocarcinoma.

Results

Liver fluke burden positively correlated with *Helicobacter* infection

The distribution of infection with *Helicobacter* spp. in regions endemic for opisthorchiasis was established according to age, gender, burden of liver fluke, as diagnosed fecal EPG and ultrasonography score for hepatobiliary disease including fibrosis. A total of 553 residents from four provinces of Thailand participated in the study; samples of feces from 260 participants were egg negative whereas 293 were positive for eggs of *O. viverrini* (Table 1).

In addition to infection with *O. viverrini*, analysis of feces by PCR was used to investigate the presence of *Helicobacter* spp. A total of 267 residents of four Isaan provinces of Thailand were positive for *H. pylori*, 99 for *H. bilis* and 18 for *H. hepaticus.* Gender did not correlate with presence of species of *Helicobacter*, $P > 0·05$ (Table 1).

The prevalence of infection with *H. pylori* assigned as *ure*A gene-positive by stool PCR was 64·6% vs. 29·6% in *O .viverrini*-infected and uninfected participants, respectively; $P < 0·01$. The prevalence of infection with *H. bilis*, but not *H. hepaticus*, was also significantly higher in *O. viverrini*-infected vs. uninfected individuals, 29·3 vs 5·4%, $P < 0·01$. In addition, mixed *H. pylori/H. bilis* infection was significantly higher during infection with *O .viverrini*: 26·9% compared to participants who were stool-negative for *O. viverrini*, 4·2%, $P < 0·01$ (Figure 1).

Increased prevalence and load of *H. pylori* and *H. bilis* during opisthorchiasis

The mass of *H. pylori* in one-gram of feces correlated positively with the intensity of liver fluke infection (one-way ANOVA, $P < 0·001$) (Supplementary Figure S1). In general, participants with higher intensity infection (>1,000 EPG) had ~15 times the total cell counts of *H. pylori* compared those who were negative for infection with *O. viverrini* (EPG = 0). Load of *H. pylori* increased according to intensity of liver fluke infection; $P < 0·001$ for each sequential comparison (Supplementary Figure S1).

The positive relationship between *Helicobacter* and *O. viverrini* infection was substantiated by positive correlations between 16S rRNA and intensity of liver fluke infection ($\chi^2 = 0·06$), *ureA* (*H. pylori*) and intensity of liver fluke infection (χ^2 trend < 0·001), *cag*A and intensity of liver fluke infection (χ^2 trend < 0·001), *ca*gE and intensity of liver fluke infection (χ^2 trend < 0·001), and *H. bilis* and intensity of liver fluke infection (χ^2 trend < 0·001); but not for *H. hepaticus*. Generally, the presence of *H. pylori* and *H. bilis* was far higher during elevated levels of infection with *O. viverrini* than during low intensity infections or in the uninfected participants. Table 2 details the findings.

Helicobacter spp. associated with grade of biliary peridcutal fibrosis

The presence of *cagA* was associated with an elevated risk of both grade 2 and grade 3 biliary periductal fibrosis. The relative risk ratio (RRR) for grade 2 versus grade 1 or 0 hepatobiliary disease was 3·38 (95% C1·51-7·58, $P = 0·003$) comparing individuals with and without *cagA*, in the model adjusted for age and sex (Table 3). The analogous RRR was 9·15 for grade 3 vs. grade

1 or 0 hepatobiliary disease (95% CI 1·74 - 47·97, *P* = 0·009) (Table 3). After confirming the proportional odds assumption, we determined and overall odds ratio of 4·24 for each subsequent grade of hepatobiliary disease comparing individuals with and without *cag*A, controlling for age and sex; *P* < 0·001.

Also, a strong, positive association was evident between the presence of mixed *cag*A and *cag*E and marked hepatobiliary disease; RRR = 4·96 for grade 3 vs. grade 1 or 0, 95% CI =1·50-16·34, *P* = 0·009. Association was not evident between positivity for mixed *cag*A and *cag*E and grade 2-biliary periductal fibrosis. Associations between the presence of *H. pylori*, *H. bilis*, *H. hepaticus* alone or in combination with hepatobiliary disease were not significant

_cag_A genotypes associated with biliary periductal fibrosis

In order to categorize the *cag*A genotypes, sequence analysis was undertaken on *cag*A-positive samples. Seventy-seven *cag*A strains were Western CagA type and unclassified type, AB type. The predominant CagA types were EPIYA-AB type, EPIYA-ABC type and EPIYA-AB'C type (B'=EPIYT)[39]. Participants who were not infected with *O. viverrini* showed higher frequency of EPIYA-AB type than did the infected participants, 86·7 % vs. 75·8%, respectively (Table 4). On the other hand, *O. viverrini*-infected participants carried a marginally higher frequency of EPYA-ABC type (8·1 vs. 6·7%) and twice as high frequency of EPIYA-AB'C (16·1 vs. 6·7%) (Table 4). In overview, the Western type CagA with EPIYA-AB'C showed higher frequency in the liver fluke-infected cases.

In addition, some *cag*A genotypes included the CagA multimerization (CM) motif. CM is comprised of 16 amino acids, FPLKRYDKFDDLSKVG or FPLKRHDKFDDLSKVG and is highly conserved for Western and Eastern CagA [40,41]. Whereas the prevalence of CagA with CM in EPIYA-AB type was 30·8% – 36·2% in liver fluke infection-negative and -positive participants, respectively, CM was present in all (100%) of the EPIYA-ABC and EPIYA-AB'C (EPIYT) genotypes detected (Table 5).

Concerning associations between CagA types and grade of biliary periductal fibrosis, significant associations between AB'C type versus AB type and both grade 2 (RRR = 23·12, 95% CI =2·31-23·50, *P* = 0·007, and grade 3 (RRR= 24·36, 95% CI = 1·71-347·09, *P* = 0·018) were apparent (Table 6). There was no association between ABC type versus AB type and hepatobiliary disease. In addition, regarding CagA types with or without the CM sequence, significant association between CagA with CM sequence and grade 2 was evident (RRR = 30·74, 95% CI = 5·25-180·08, *P* < 0·001). After confirming the proportional odds assumption, we determined an overall odds ratio of 30·8 for each subsequent grade of biliary periductal fibrosis comparing individuals carrying CagA with and without the CM sequence, and controlling for age and sex (*P* < 0·001). Similarly, after grouping the degree of hepatobiliary disease as either negative (grades 0+1) or positive (grades 2+3), as described [42,43], a significant association was apparent between positive for hepatobiliary disease and CagA with CM sequence with an odds ratio of 38·21 (95% CI = 6·85 - 213·03, *P* < 0·001). On the other hand, the wide range for CI in this analysis reflected the limited number of cases in the dataset due to this uncommon genotype and, in turn, the limited power of this analysis.

Phylogram analysis of CagA EPIYA motifs revealed novel genotypes during liver fluke infection

The phylogenetic relationships of CagA genotypes among 75 samples from this cohort of participants from northeastern Thailand, specifically 13 negative and 62 positive for infection with *O. viverrini*, were compared with two Western *cag*A and two Eastern *cag*A reference strains detected in gastro-duodenal disease in Thailand, and three CagA sequences isolated from bile from Thai cholangiocarcinoma (CCA) cases, as reported[33,44]. The relationships were determined using maximum parsimony. Representative *cag*A-encoded sequences of our Thai cohort mainly grouped into main clusters: 1) Unclassified type, EPIYA AB without CM sequence, e.g. samples FPNS105, FNK3; 2) Unclassified type, EPIYA AB with CM sequence, e.g. FNK9, FNK10; 3) 'Western-like' type EPIYA ABC, e.g. FPK3, FPNS5; and 4) 'Western-like' type EPIYA AB'C, e.g. FPK4, FPNS8. By contrast, the sequences of Western, Eastern and CCA cases grouped together, generally divergent from sequences in the present cohort (Figure 2). The association between the 'Western like' genotypes, including EPIYA AB'C, and the hepatobiliary pathology is evident for the sequences analyzed in the phylogenetic tree shown in Figure 2A. Figure 2B depicts representative sequences that belong to the main clusters of CagA described above, indicating the EPIYA motives and CM sequences. Whereas the prevalence of *cag*A-encoding the CM sequence in EPIYA-AB type was 35 %, CM was present in all (100%) of the EPIYA-ABC and EPIYA-AB'C (EPIYT) genotypes characterized here (Table 5).

Discussion

County-wide sampling indicates a prevalence of carriage of *H. pylori* by asymptomatic Thais of ~ 44%, based on fecal examination [41], with marginally higher sero-prevalence [45]. This report describes an association between infection with the fish-borne liver fluke *O. viverrini* and carriage of species of *Helicobacter* in opisthorchiasis-endemic northeastern Thailand. *H. pylori* represented the major species of *Helicobacter* but, in addition, *H. bilis* and mixed *H. pylori/H. bilis* infection occurred more often during active opisthorchiasis than in uninfected or lightly infected persons, in turn confirming earlier reports [46,47]. Mixed infection with *H. pylori* and *H. bilis* may be associated with more severe hepatobiliary disease. Prevalence of *H. pylori* and *H. bilis* also was elevated in participants who were heavily infected with *O. viverrini*. Opisthorchiasis appeared to exacerbate severity of *H. pylori*/ *H. bilis*-associated disease in like fashion to infections in hamsters [35,37], confirming an association between intensity of *H. pylori*/ *H. bilis* infection and presence of the liver fluke.

Prevalence of both *cagA*-and *cagE*-positive *H. pylori* positively correlated with increasing levels of liver fluke infection, and prevalence of *cag*A-positive strains of *H. pylori* correlated positively with increased biliary periductal fibrosis as diagnosed by abdominal ultrasound. The presence of *cag*A- and *cag*E-positive *H. pylori* strains associated with severe fibrosis, findings that suggested that *H. pylori*, and in particular *cag*A-positive strains, reached the biliary tract, and induced hepatic inflammation that exacerbated periductal fibrosis. Discrete genotypes of *cag*A associate with severity of gastrointestinal diseases [48]. Unclassified type (AB type) represented the major *cag*A genotype in this study, in contrast with earlier reports indicating that AB represents only a minority genotype carried by otherwise healthy Thais [41,49]. Here, 22% of the *cag*A-encoded sequences were Western type (ABC type) with no East Asian type (ABD type), lower than reported for liver fluke infection-induced CCA [44]. A meta-analysis of *cag*A status in Southeast

Asia has revealed 51% vs. 49% of Western type and East Asian type, respectively [48]. There was a higher prevalence of typical Western type (EPIYA ABC) and variant AB'C type (EPIYT B) *cag*A genotypes in *O. viverrini*-infected compared to uninfected participants.

The present findings also demonstrated that polymorphisms in *cag*A of *H. pylori* circulate among Thais with opisthorchiasis. For the ABC and AB'C type CagA, there was a higher frequency of the deduced 16-amino-acid CagA multimerization (CM) types during liver fluke infection. CM is conserved between Western CagA and East Asian CagA [50], although Western type CagA invariably exhibits the CM sequence [39]. The CM sequence represents a membrane-targeting signal [50], which interacts with PAR1b, thus inducing junctional and polarity defects [29,50,51]. Notably, the PCR primers employed here spanned the entire 3'-region of *cagA* encoding the multimers of the tyrosine phosphorylation motifs[52,53]. Structural polymorphism in the CM reflects the degree of virulence of CagA [54]. Here infection with any CagA type *H. pylori* bearing CM sequences was associated with severe hepatobiliary disease, with an odds ratio up as high as 38. This characterization of sequences with both EPIYA-C/D motif and CM sequence suggested increased phosphorylation motifs capable of provoking pronounced disease [54]. Phylogenetic analysis revealed four discrete clades, and all four differed from the from typical Western and East Asian CagA types including those associating with Western CCA sequences [44]. Although the Thai CagA sequences were separated from the pathogenic reference sequences, opisthorchiasis might be involved in the various novel types of CagA (with CM sequence), which associates with severe disease. Accordingly, we hypothesize that not only is the liver fluke *O. viverrini* a reservoir of *Helicobacter* but also a selector for pathogenic strains of this ε-proteobacterium. Given the elevated presence of *H. pylori*, and CagA including its polymorphisms with increasing intensity of liver fluke infection and biliary tract fibrosis, these new variants may, at least partly, underlie progression of hepatobiliary disease in opisthorchiasis-endemic regions.

The International Agency for Research on Cancer of the World Health Organization classifies infection with the liver flukes *O. viverrini* and *Clonorchis sinensis* and with *H. pylori* as Group 1 carcinogens[4]. In northern and northeastern Thailand and Laos, infection with *O. viverrini* is the major risk for CCA [4,8,55,56]. Following initiation, oncogenesis appears to be promoted by cholestasis and chronic inflammation. Increased mutation rates of the tumor suppressor genes *p53* and *CDKN2A*, and of genes encoding protein tyrosine phosphatases, SMAD4 and others sustain cholangio-carcinogenesis, with differences between CCA induced by opisthorchiasis compared to other risks factors[57,58]. As reviewed [59], the release and interaction of interleukin-6, transforming growth factor beta, tumor necrosis factor alpha, and platelet-derived growth factor are pivotal to the proliferation of cholangiocytes, while evasion of apoptosis, autonomous proliferation, and angiogenesis sustain incipient neoplasia. In parallel, infection with *cagA*-positive *H. pylori* is the major risk for gastric adenocarcinoma and mucosa associated lymphoid tissue (MALT) lymphoma. Cellular changes following the injection of the CagA oncoprotein include epithelial to mesenchymal transition and the hummingbird phenotype[60,61], along with genetic mutations in E-cadherin and epigenetic changes. Genome sequencing has identified driver mutations TP53, ARID1A, CDH1, MUC6, CTNNA2, GLI3, RNF43 and others in gastric cancer [62]. Loss of epithelial cadherin expression from *CDH1* alterations is a primary carcinogenetic incident. Cytogenetic abnormalities including the t(11;18)(q21;q21) translocation are frequently acquired during *H. pylori*-associated gastric MALT lymphoma [63].

The association between opisthorchiasis and the presence of *H. pylori* in feces was statistically significant. Nonetheless, direct evidence of a causal relationship where *H. pylori* and liver fluke infection jointly prime the pathogenesis of hepatobiliary disease including CCA has not been obtained. It is relevant to note the outcome of a recent study using a rodent model of human opisthorchiasis, which provides support for the association among *O. viverrini*, *H. pylori* and biliary periductal fibrosis [37,64]. Liver fluke-infected hamsters were treated with antibiotics and the anthelmintic, praziquantel. Quantitative PRC analysis of tissue and organs from the hamsters indicated that the majority of the *H. pylori* emanated from the same sites as the liver flukes in the biliary tract given that antibiotics failed to reduce the load of *H. pylori* to the baseline achieved with dual treatment with antibiotics and praziquantel. *H. pylori* load in the stomach was unaffected. In addition, immunohistochemical approaches detected *H. pylori* within the gut of liver flukes recovered from the hamsters.

Hepatobiliary disorders caused by *Helicobacter*[33,44,65] can resemble opisthorchiasis [42,66]. Chronic lesions ascribed to liver fluke infection, including cholangitis, biliary hyperplasia and metaplasia, periductal fibrosis and CCA, may be due in part to *Helicobacter*-associated hepatobiliary disease. *H. pylori* DNA has been isolated from tissues from CCA and from cholecystitis/cholelithiasis in regions endemic for opisthorchiasis [33,44]. Moreover, serological findings indicate infection with *H. pylori* in Thais at high risk for CCA [65]. An explanation for why infection with the liver fluke induces bile duct cancer [10] might now be clearer – involvement by *H. pylori* and its virulence factors. The spiral bacilli of *H. pylori* attach to biliary cells, which internalize in similar fashion to their behavior on gastric epithelium [29,67]. *Helicobacter* likely passes from the stomach to the duodenum and enters the biliary tree through the duodenal papilla and ampulla of Vater [40,67]. How the microbe tolerates the neutral to alkaline pH of the small intestine and biliary tree remains unclear [17]. However, an association with the migrating liver flukes offers a plausible explanation: given that *Helicobacter*-like curved rods occur in the gut of *O. viverrini* [37], and given that the micro-environment of the *O. viverrini* gut is acidic, the microbe might hitchhike within the migrating juvenile trematode. Intriguingly, glycoprotein gylcans expressed on the gut epithelium of *O. viverrini* [68] resemble receptors of gastric epithelial cells to which *H. pylori* binds [69]. *Helicobacter* may have evolved a commensalism with *O. viverrini*, with conveyance into the biliary tract during the migration of the parasite following ingestion of the metacercaria with undercooked freshwater fish [35,37].

Given the elevated prevalence of CCA in regions where infection with liver fluke prevails, and given the increasing evidence of linkage between carriage of *Helicobacter* during opisthorchiasis, these two biological carcinogens together may orchestrate the pathogenesis of opisthorchiasis and bile duct cancer. The association of *Helicobacter* and its virulence factors, together with chronic opisthorchiasis, may underlie biliary tract disease including CCA in liver fluke-endemic regions [70]. Whereas additional studies are needed to clarify this association, at present detection of *H. pylori* in feces provides a non-invasive approach to investigate its association with biliary tract disease during opisthorchiasis.

Materials and Methods

Ethics statement

The Institutional Human Ethics Committee of Khon Kaen University approved the study, approval number HE 551332. All methods were performed in accordance with the relevant guidelines and regulations of the committee. The participants provided written informed consent following discussion with the researchers that included information on fecal samples for laboratory analyses. All participants were adults; children were not enrolled (Table 1).

Study participants

Participants were asked to refrain for up to 10 days from consumption of fatty foods, antacid medication, antibiotics, anti-parasitic agents, barium, mineral oil, bismuth, or non-absorbable anti-diarrheal agents. Patients with history of digestive-tract diseases (gastritis, gastric ulcer, cholecystitis, cholangitis, cholecystectomy, others) were excluded from the study. A total of 553 participants provided stool samples; 260 were parasitologically negative for fecal eggs of *O. viverrini* and 293 were egg-positive for *O. viverrini* from age-sex matched residents of villages in four provinces of the opisthorchiasis-endemic Isaan region of northeastern Thailand[1,8,42,71]. In particular, those enrolled included 273, 107, 93 and 80 people from the provinces of Khon Kaen, Roi-et, Mahasarakham and Kalasin, respectively (Supplementary Figure S2). The participants included 288 females and 265 males, aged 30 to 70 years (Table 1).

Parasitological diagnosis of infection with the liver fluke *Opisthorchis viverrini*

Parasitological diagnosis of opisthorchiasis was accomplished using formalin-ethyl acetate concentration of one gram of feces, followed by light microscopy examination of the concentrate [72]. The method is suitable for diagnosis of *O. viverrini* eggs and widely employed for diagnosis of opisthorchiasis [72]. Thereafter, participants were grouped according to fecal egg count, i.e. intensity of infection into five categories: 1) EPG (eggs per gram of feces) = 0 [i.e. uninfected]; 2) 1-100 EPG; 3) 101-500 EPG; 4) 501-1,000 EPG; and 5) >1,000 EPG. There were 260, 193, 73, 12 and 15 participants in these five categories, respectively (Table 2).

Detection by PCR of *Helicobacter* species and virulence genes

DNA was isolated from about one gram of feces, stored in 70% ethanol, using a QIAamp DNA Stool Mini Kit (Qiagen, Germany) with concentrations ranging from 50 to 500 ng/µl, and total yields of 2 to 15 µg. Subsequently, 50 ng DNA from the samples served as the template for PCR performed in a GeneAmp PCR system 9700, Applied Biosystems thermal cycler; the reaction mixture included 1x Gotaq Colorless Master Mix (Promega) containing 0·2 mM dNTP, 1·5 mM $MgCl_2$, 1·25 U *Tag* DNA polymerase, with primers at 0·2 mM each. Supplementary Table S1 provides the gene specific primers for *Helicobacter* species, specifically for 16S rRNA, *ure*A, *cagA*, *cagE* of *H. pylori*, and for *H. bilis* and *H. hepaticus*. Amplicons were sized by electrophoresis through 1·0 % agarose, stained with ethidium bromide and visualized under UV light. The expected sizes of amplicons for the 16S rRNA, *ure*A *(H. pylori*), *H. bilis, H. hepaticus, cagA* sequencing and *cagE* were 480, 350, 418, 405, 550-800, and 508 bp, respectively (Supplementary Figure S3).

Abdominal ultrasonography to visualize hepatobiliary fibrosis

Abdominal scans were performed using a mobile high-resolution ultrasound-imaging appliance (GE model LOGIQ Book XP), as described [43,73]. Hepatobiliary abnormalities including periductal fibrosis in liver parenchyma, gallbladder wall, gallbladder size, sludge, and suspected CCA (dilated intra or extrahepatic bile duct and/or liver mass) were graded and recorded [4234]. Based on the ultrasonography, grading of periductal biliary fibrosis was assigned as follows: grade 0 = absence of periportal echo(s) from all segments of liver; grade 1 = presence of periportal echo(s) in one segment of liver; grade 2 = periportal echo(s) in two to three segments; grade 3 = periportal echo(s) in more than three segments. Status of infection with liver fluke or presence of species of *Helicobacter* was not known by the radiologist during the abdominal ultrasonography.

Quantitative real-time PCR

Fecal samples from the *H. pylori* infected (conventional PCR *ure*A-positive) participants (n = 267) used in this study were assigned to one of five groups based on fecal EPG for *O. viverrini* (above): *O. viverrini* EPG = 0 (n = 77), EPG = 1-100 (n = 110), EPG = 101- 500 (n = 57), EPG = 501-1,000 (n = 10) and EPG > 1,000 (n = 13). In addition, feces free of *H. pylori* were included as a negative control [37]. Presence of *H. pylori* was established and quantified by real time PCR using primers HpyF1: GGGTATTGAAGCGATGTTTCCT and HpyR1: GCTTTTTTGC-CTTCGTTGATAGT[44]. The quantitative real-time analysis targeted the species-specific gene *ure*A of *H. pylori* [74]. DNA samples were diluted to employ equivalent template concentrations in the qPCR reactions that included 10 µl SYBR master mix (Thermo Fisher), 1 µl template-DNA, 0·5 µl of each primer (625 nM), and 9 µl nuclease-free water. PCR was performed in triplicate (technical triplicates) in a thermal cycler (Light Cycler 1·5, Roche), using initial denaturation at $95^{\circ}C$ for 9 min, followed by 40 cycles of $95^{\circ}C$, 15 s, $60^{\circ}C$, 60 s for the annealing and elongation steps, respectively. A 10-fold serial dilution of *H. pylori* DNA was included to establish a standard curve, from 10^8 cells/ml to 10^1 cells/ml; bacterial cells were counted in a Thoma-counting-chamber, plated and incubated for subsequent extraction of DNA. *E. coli* DNA served as the negative control [74].

Phylogenetic analysis of *cag*A gene partial sequences

To establish phylogenetic relationships among the *H. pylori* genotypes, 62 participants infected and 13 uninfected with *O. viverrini* were investigated. Partial sequences of *cag*A genes amplified by PCR were sequenced by the Sanger approach (First BASE Laboratories, Malaysia). In addition, sequences of Western-like CagA from four references were analyzed: Thailand (GenBank accession BAB87427 [75]) Western, Thailand (BAB87428) Western, Thailand (BAB87429) eastern, and Thailand (BAB87430) eastern. Partial, deduced amino acid sequences of CagA were searched for EPIYA motifs [39,44] using the ExPASy-Translate software followed by multiple sequence alignment using ClustalW (Bioedit)[76] with further editing using GeneDoc (http://www.nrbsc.org/gfx/genedoc/ebinet.htm). Evolutionary history was inferred using Neighbor-Joining [77]. A bootstrapped consensus tree inferred from 500 replicates was taken to represent the evolutionary history of the taxa analyzed. Branches corresponding to partitions reproduced in less than 50% bootstrap replicates were collapsed. Evolutionary distances were

computed using the JTT matrix-based method [78] and the units represent the number of amino acid substitutions per site. The analysis of *cag*A included 82 deduced amino acid residues; positions containing gaps or missing data were eliminated, leaving 61 positions in the final dataset. Phylogenetic analyses were conducted with MEGA5 [79].

Statistical analysis

Both univariate and multivariate analyses were employed. Participants were categorized according to the intensity of infection with *O. viverrini*, i.e. EPG = 0, 1 to 100, 101 to 500, 501 to 1,000, and >1,000. The findings are presented in a box and whisker plot, and means of total bacterial cell counts per gram of feces according to intensity of infection with *O. viverrini* were compared using a one-way analysis of variance (ANOVA) post hoc test.

χ^2 tests were performed to determine the relationship between intensity of infection with *O. viverrini* and prevalence of *Helicobacter.* Measures of *Helicobacter* infection included PCR-positivity for the presence of the 16S rRNA gene, *ure*A, *cag*A, *cag*A genotype, *cag*E, mixed *cag*A and *cag*E, *H. bilis*, *H. hepaticus*, and *H. pylori* + *H. hepaticus.* χ^2 tests for trend were used to investigate the effect of increasing level of liver fluke infection and each parameter of infection with species of *Helicobacter*.

Age and sex adjusted relative risk ratios (RRR) and 95% confidence intervals (CIs) for presence or absence of *Helicobacter* infection, and association with hepatobiliary disease were determined using age and sex adjusted multinomial logistic regression analyses. Ordinal logistic regression was performed to determine overall odds ratios for each model; these were only presented if the proportional odds assumption was met for a given mode. Statistical tests were two-sided, and were performed using IBM SPSS Statistics, IBM Corp., NY, 2x2 Contingency Table online calculator, VassarStats, and STATA version 10, College Station, TX. $P \leq 0{\cdot}05$ was considered statistically significant.

Competing Financial Interests

The authors declare there were no competing financial interests.

Acknowledgments

We thank Dr. Apiporn Thinkhamrop, Khon Kaen University for assistance in preparation of map of the study sites, and Dr. Makedonka Mitreva and laboratory colleagues for comments on the study findings. We acknowledge the advice of Dr. Supot Kamsa-ard, Khon Kaen University and Dr. Isha Agarwal, Harvard University for statistical analysis. R.D. acknowledges support as a PhD research scholar from the Commission on Higher Education, Thailand, under the program Strategic Scholarships for Frontier Research Network for the Joint PhD Program Thai Doctoral Degree; B.S. acknowledges support from Thailand Research Fund Senior Research Scholar; and A.L. acknowledges support from his NHMRC Principal Research Fellowship. This work was supported by the National Health Security Office, Thailand, the Higher Education Research Promotion and National Research University Project of Thailand, Office of the Higher Education Commission, through the Health Cluster (SHeP-GMS), the Faculty of Medicine, Khon Kaen

University, Thailand (award number I56110), and the Thailand Research Fund under the TRF Senior Research Scholar (RTA 5680006); the National Research Council of Thailand. The National Institute of Allergy and Infectious Diseases (NIAID), Tropical Medicine Research Center award number P50AI098639, The National Cancer Institute, award number R01CA164719, and the United States Army Medical Research and Materiel Command (USAMRMC), contract number W81XWH-12-C-0267 also provided support. The content is solely the responsibility of the authors and does not necessarily represent the official views of the funders including USAMRMC, NIAID, NCI or the NIH.

Author Contributions Statement

B.S., C.C., R.D., C.P., Y.C., and P.J.B. conceived and designed the study. B.S., E.M., and P.M. collected stool samples, demographic and ultrasonographic data. R.D., B.S., and C.C. performed the experiments. G.R., P.J.B., R.D. and B.S. analyzed and interpreted the phylogenetic findings. B.S., R.D., C.C., A.L. and P.J.B. analyzed and interpreted overall data. B.S., R.D., G.R., C.C., A.L., and P.J.B. wrote the manuscript. All authors read and approved the final version of the paper.

References

1 Petney, T. N., Andrews, R. H., Saijuntha, W., Wenz-Mucke, A. & Sithithaworn, P. The zoonotic, fish-borne liver flukes Clonorchis sinensis, Opisthorchis felineus and Opisthorchis viverrini. *Int J Parasitol* **43**, 1031-1046, doi:10.1016/j.ijpara.2013.07.007 (2013).

2 Sripa, B., Kaewkes, S., Intapan, P. M., Maleewong, W. & Brindley, P. J. Food-borne trematodiases in Southeast Asia epidemiology, pathology, clinical manifestation and control. *Adv Parasitol* **72**, 305-350, doi:10.1016/S0065-308X(10)72011-X (2010).

3 Sithithaworn, P. *et al.* The current status of opisthorchiasis and clonorchiasis in the Mekong Basin. *Parasitol Int* **61**, 10-16, doi:10.1016/j.parint.2011.08.014 (2012).

4 Humans, I. W. G. o. t. E. o. C. R. t. Biological agents. Volume 100 B. A review of human carcinogens. *IARC Monogr Eval Carcinog Risks Hum* **100**, 1-441 (2012).

5 Sripa, B. *et al.* Liver fluke induces cholangiocarcinoma. *PLoS Med* **4**, e201, doi:10.1371/journal.pmed.0040201 (2007).

6 Bouvard, V. *et al.* A review of human carcinogens--Part B: biological agents. *Lancet Oncol* **10**, 321-322 (2009).

7 Sripa, B. *et al.* The tumorigenic liver fluke Opisthorchis viverrini--multiple pathways to cancer. *Trends Parasitol* **28**, 395-407, doi:10.1016/j.pt.2012.07.006 (2012).

8 Khuntikeo, N., Loilome, W., Thinkhamrop, B., Chamadol, N. & Yongvanit, P. A Comprehensive Public Health Conceptual Framework and Strategy to Effectively Combat Cholangiocarcinoma in Thailand. *PLoS Negl Trop Dis* **10**, e0004293, doi:10.1371/journal.pntd.0004293 (2016).

9 Sripa, B. Pathobiology of opisthorchiasis: an update. *Acta Trop* **88**, 209-220 (2003).

10 Jurberg, A. D. & Brindley, P. J. Gene function in schistosomes: recent advances toward a cure. *Front Genet* **6**, 144, doi:10.3389/fgene.2015.00144 (2015).

11 Jusakul, A., Yongvanit, P., Loilome, W., Namwat, N. & Kuver, R. Mechanisms of oxysterol-induced carcinogenesis. *Lipids Health Dis* **10**, 44, doi:10.1186/1476-511X-10-44 (2011).
12 Correia da Costa, J. M. *et al.* Schistosome and liver fluke derived catechol-estrogens and helminth associated cancers. *Front Genet* **5**, 444, doi:10.3389/fgene.2014.00444 (2014).
13 Abu Al-Soud, W. *et al.* DNA of Helicobacter spp. and common gut bacteria in primary liver carcinoma. *Dig Liver Dis* **40**, 126-131, doi:10.1016/j.dld.2007.09.011 (2008).
14 Flahou, B., Rimbara, E., Mori, S., Haesebrouck, F. & Shibayama, K. The Other Helicobacters. *Helicobacter* **20 Suppl 1**, 62-67, doi:10.1111/hel.12259 (2015).
15 Cover, T. L. Helicobacter pylori Diversity and Gastric Cancer Risk. *MBio* **7**, e01869-01815, doi:10.1128/mBio.01869-15 (2016).
16 Marshall, B. J. The pathogenesis of non-ulcer dyspepsia. *Med J Aust* **143**, 319 (1985).
17 Gaynor, E. C. & Szymanski, C. M. The 30th anniversary of Campylobacter, Helicobacter, and Related Organisms workshops-what have we learned in three decades? *Front Cell Infect Microbiol* **2**, 20, doi:10.3389/fcimb.2012.00020 (2012).
18 Sheh, A. & Fox, J. G. The role of the gastrointestinal microbiome in Helicobacter pylori pathogenesis. *Gut Microbes* **4**, 505-531, doi:10.4161/gmic.26205 (2013).
19 Marshall, B. J. & Warren, J. R. Unidentified curved bacilli in the stomach of patients with gastritis and peptic ulceration. *Lancet* **1**, 1311-1315 (1984).
20 Moodley, Y. *et al.* Age of the association between Helicobacter pylori and man. *PLoS Pathog* **8**, e1002693, doi:10.1371/journal.ppat.1002693 (2012).
21 Kienesberger, S. *et al.* Gastric Helicobacter pylori Infection Affects Local and Distant Microbial Populations and Host Responses. *Cell Rep* **14**, 1395-1407, doi:10.1016/j.celrep.2016.01.017 (2016).
22 Cover, T. L. & Blaser, M. J. Helicobacter pylori in health and disease. *Gastroenterology* **136**, 1863-1873, doi:10.1053/j.gastro.2009.01.073 (2009).
23 de Martel, C., Plummer, M., Parsonnet, J., van Doorn, L. J. & Franceschi, S. Helicobacter species in cancers of the gallbladder and extrahepatic biliary tract. *Br J Cancer* **100**, 194-199, doi:10.1038/sj.bjc.6604780 (2009).
24 Fallone, C. A. *et al.* Helicobacter DNA in bile: correlation with hepato-biliary diseases. *Aliment Pharmacol Ther* **17**, 453-458 (2003).
25 Apostolov, E. *et al.* Helicobacter pylori and other Helicobacter species in gallbladder and liver of patients with chronic cholecystitis detected by immunological and molecular methods. *Scand J Gastroenterol* **40**, 96-102 (2005).
26 Kobayashi, T., Harada, K., Miwa, K. & Nakanuma, Y. Helicobacter genus DNA fragments are commonly detectable in bile from patients with extrahepatic biliary diseases and associated with their pathogenesis. *Dig Dis Sci* **50**, 862-867 (2005).
27 Kosaka, T. *et al.* Helicobacter bilis colonization of the biliary system in patients with pancreaticobiliary maljunction. *Br J Surg* **97**, 544-549, doi:10.1002/bjs.6907 (2010).
28 Aviles-Jimenez, F. *et al.* Microbiota studies in the bile duct strongly suggest a role for Helicobacter pylori in extrahepatic cholangiocarcinoma. *Clin Microbiol Infect* **22**, 178 e111-122, doi:10.1016/j.cmi.2015.10.008 (2016).
29 Hatakeyama, M. Helicobacter pylori CagA and gastric cancer: a paradigm for hit-and-run carcinogenesis. *Cell Host Microbe* **15**, 306-316, doi:10.1016/j.chom.2014.02.008 (2014).
30 Mateos-Munoz, B. *et al.* Enterohepatic Helicobacter other than Helicobacter pylori. *Rev Esp Enferm Dig* **105**, 477-484 (2013).

31 Zhou, D. *et al.* Infections of Helicobacter spp. in the biliary system are associated with biliary tract cancer: a meta-analysis. *Eur J Gastroenterol Hepatol* **25**, 447-454, doi:10.1097/MEG.0b013e32835c0362 (2013).
32 Murphy, G. *et al.* Association of seropositivity to Helicobacter species and biliary tract cancer in the ATBC study. *Hepatology* **60**, 1963-1971, doi:10.1002/hep.27193 (2014).
33 Boonyanugomol, W. *et al.* Helicobacter pylori in Thai patients with cholangiocarcinoma and its association with biliary inflammation and proliferation. *HPB (Oxford)* **14**, 177-184, doi:10.1111/j.1477-2574.2011.00423.x (2012).
34 Carpenter, H. A. Bacterial and parasitic cholangitis. *Mayo Clin Proc* **73**, 473-478, doi:10.1016/S0025-6196(11)63734-8 (1998).
35 Plieskatt, J. L. *et al.* Infection with the carcinogenic liver fluke Opisthorchis viverrini modifies intestinal and biliary microbiome. *FASEB J* **27**, 4572-4584, doi:10.1096/fj.13-232751 (2013).
36 Greiman, S. E., Rikihisa, Y., Cain, J., Vaughan, J. A. & Tkach, V. V. Germs within Worms: Localization of Neorickettsia sp. within Life Cycle Stages of the Digenean Plagiorchis elegans. *Appl Environ Microbiol* **82**, 2356-2362, doi:10.1128/AEM.04098-15 (2016).
37 Deenonpoe, R. *et al.* The carcinogenic liver fluke Opisthorchis viverrini is a reservoir for species of Helicobacter. *Asian Pac J Cancer Prev* **16**, 1751-1758 (2015).
38 Saltykova, I. V. *et al.* Biliary Microbiota, Gallstone Disease and Infection with Opisthorchis felineus. *PLoS Negl Trop Dis* **10**, e0004809, doi:10.1371/journal.pntd.0004809 (2016).
39 Xia, Y., Yamaoka, Y., Zhu, Q., Matha, I. & Gao, X. A comprehensive sequence and disease correlation analyses for the C-terminal region of CagA protein of Helicobacter pylori. *PloS one* **4**, e7736, doi:10.1371/journal.pone.0007736 (2009).
40 Pellicano, R., Menard, A., Rizzetto, M. & Megraud, F. Helicobacter species and liver diseases: association or causation? *Lancet Infect Dis* **8**, 254-260, doi:10.1016/S1473-3099(08)70066-5 (2008).
41 Hirai, I. *et al.* Infection of less virulent Helicobacter pylori strains in asymptomatic healthy individuals in Thailand as a potential contributing factor to the Asian enigma. *Microbes Infect* **12**, 227-230, doi:10.1016/j.micinf.2009.12.007 (2010).
42 Mairiang, E. *et al.* Ultrasonography assessment of hepatobiliary abnormalities in 3359 subjects with Opisthorchis viverrini infection in endemic areas of Thailand. *Parasitol Int* **61**, 208-211, doi:10.1016/j.parint.2011.07.009 (2012).
43 Sripa, B. *et al.* Advanced periductal fibrosis from infection with the carcinogenic human liver fluke Opisthorchis viverrini correlates with elevated levels of interleukin-6. *Hepatology* **50**, 1273-1281, doi:10.1002/hep.23134 (2009).
44 Boonyanugomol, W. *et al.* Molecular analysis of Helicobacter pylori virulent-associated genes in hepatobiliary patients. *HPB (Oxford)* **14**, 754-763, doi:10.1111/j.1477-2574.2012.00533.x (2012).
45 Fock, K. M. & Ang, T. L. Epidemiology of Helicobacter pylori infection and gastric cancer in Asia. *J Gastroenterol Hepatol* **25**, 479-486, doi:10.1111/j.1440-1746.2009.06188.x (2010).
46 Bulajic, M. *et al.* Helicobacter pylori and the risk of benign and malignant biliary tract disease. *Cancer* **95**, 1946-1953, doi:10.1002/cncr.10893 (2002).

47 Matsukura, N. *et al.* Association between Helicobacter bilis in bile and biliary tract malignancies: H. bilis in bile from Japanese and Thai patients with benign and malignant diseases in the biliary tract. *Jpn J Cancer Res* **93**, 842-847 (2002).
48 Sahara, S. *et al.* Role of Helicobacter pylori cagA EPIYA motif and vacA genotypes for the development of gastrointestinal diseases in Southeast Asian countries: a meta-analysis. *BMC Infect Dis* **12**, 223, doi:10.1186/1471-2334-12-223 (2012).
49 Hirai, I., Yoshinaga, A., Kimoto, A., Sasaki, T. & Yamamoto, Y. Sequence analysis of East Asian cagA of Helicobacter pylori isolated from asymptomatic healthy Japanese and Thai individuals. *Curr Microbiol* **62**, 855-860, doi:10.1007/s00284-010-9797-9 (2011).
50 Murata-Kamiya, N. Pathophysiological functions of the CagA oncoprotein during infection by Helicobacter pylori. *Microbes Infect* **13**, 799-807, doi:10.1016/j.micinf.2011.03.011 (2011).
51 Hashi, K. *et al.* Natural variant of the Helicobacter pylori CagA oncoprotein that lost the ability to interact with PAR1. *Cancer Sci* **105**, 245-251, doi:10.1111/cas.12342 (2014).
52 Rudi, J. *et al.* Diversity of Helicobacter pylori vacA and cagA genes and relationship to VacA and CagA protein expression, cytotoxin production, and associated diseases. *J Clin Microbiol* **36**, 944-948 (1998).
53 Argent, R. H., Zhang, Y. & Atherton, J. C. Simple method for determination of the number of Helicobacter pylori CagA variable-region EPIYA tyrosine phosphorylation motifs by PCR. *J Clin Microbiol* **43**, 791-795, doi:10.1128/JCM.43.2.791-795.2005 (2005).
54 Lu, H. S. *et al.* Structural and functional diversity in the PAR1b/MARK2-binding region of Helicobacter pylori CagA. *Cancer Sci* **99**, 2004-2011, doi:10.1111/j.1349-7006.2008.00950.x (2008).
55 Sungkasubun, P. *et al.* Ultrasound screening for cholangiocarcinoma could detect premalignant lesions and early-stage diseases with survival benefits: a population-based prospective study of 4,225 subjects in an endemic area. *BMC Cancer* **16**, 346, doi:10.1186/s12885-016-2390-2 (2016).
56 Aye Soukhathammavong, P. *et al.* Subtle to severe hepatobiliary morbidity in Opisthorchis viverrini endemic settings in southern Laos. *Acta Trop* **141**, 303-309, doi:10.1016/j.actatropica.2014.09.014 (2015).
57 Chan-On, W. *et al.* Exome sequencing identifies distinct mutational patterns in liver fluke-related and non-infection-related bile duct cancers. *Nat Genet* **45**, 1474-1478, doi:10.1038/ng.2806 (2013).
58 Gao, Q. *et al.* Activating mutations in PTPN3 promote cholangiocarcinoma cell proliferation and migration and are associated with tumor recurrence in patients. *Gastroenterology* **146**, 1397-1407, doi:10.1053/j.gastro.2014.01.062 (2014).
59 Al-Bahrani, R., Abuetabh, Y., Zeitouni, N. & Sergi, C. Cholangiocarcinoma: risk factors, environmental influences and oncogenesis. *Ann Clin Lab Sci* **43**, 195-210 (2013).
60 Segal, E. D., Cha, J., Lo, J., Falkow, S. & Tompkins, L. S. Altered states: involvement of phosphorylated CagA in the induction of host cellular growth changes by Helicobacter pylori. *Proc Natl Acad Sci U S A* **96**, 14559-14564 (1999).
61 Saadat, I. *et al.* Helicobacter pylori CagA targets PAR1/MARK kinase to disrupt epithelial cell polarity. *Nature* **447**, 330-333, doi:10.1038/nature05765 (2007).

62 Wang, K. *et al.* Whole-genome sequencing and comprehensive molecular profiling identify new driver mutations in gastric cancer. *Nat Genet* **46**, 573-582, doi:10.1038/ng.2983 (2014).
63 Nie, Z. *et al.* Conversion of the LIMA1 tumour suppressor into an oncogenic LMO-like protein by API2-MALT1 in MALT lymphoma. *Nat Commun* **6**, 5908, doi:10.1038/ncomms6908 (2015).
64 Sripa, B., Deenonpoe, R. & Brindley, P. J. Co-infections with liver fluke and Helicobacter species: A paradigm change in pathogenesis of opisthorchiasis and cholangiocarcinoma? *Parasitol Int*, doi:10.1016/j.parint.2016.11.016 (2016).
65 Pisani, P. *et al.* Cross-reactivity between immune responses to Helicobacter bilis and Helicobacter pylori in a population in Thailand at high risk of developing cholangiocarcinoma. *Clin Vaccine Immunol* **15**, 1363-1368, doi:10.1128/CVI.00132-08 (2008).
66 Lvova, M. N. *et al.* Comparative histopathology of Opisthorchis felineus and Opisthorchis viverrini in a hamster model: an implication of high pathogenicity of the European liver fluke. *Parasitol Int* **61**, 167-172, doi:10.1016/j.parint.2011.08.005 (2012).
67 Boonyanugomol, W. *et al.* Helicobacter pylori cag pathogenicity island (cagPAI) involved in bacterial internalization and IL-8 induced responses via NOD1- and MyD88-dependent mechanisms in human biliary epithelial cells. *PLoS One* **8**, e77358, doi:10.1371/journal.pone.0077358 (2013).
68 Talabnin, K. *et al.* Stage-specific expression and antigenicity of glycoprotein glycans isolated from the human liver fluke, Opisthorchis viverrini. *Int J Parasitol* **43**, 37-50, doi:10.1016/j.ijpara.2012.10.013 (2013).
69 Hanisch, F. G., Bonar, D., Schloerer, N. & Schroten, H. Human trefoil factor 2 is a lectin that binds alpha-GlcNAc-capped mucin glycans with antibiotic activity against Helicobacter pylori. *J Biol Chem* **289**, 27363-27375, doi:10.1074/jbc.M114.597757 (2014).
70 Segura-Lopez, F. K., Guitron-Cantu, A. & Torres, J. Association between Helicobacter spp. infections and hepatobiliary malignancies: a review. *World J Gastroenterol* **21**, 1414-1423, doi:10.3748/wjg.v21.i5.1414 (2015).
71 Grundy-Warr, C. *et al.* Raw attitudes, wetland cultures, life-cycles: socio-cultural dynamics relating to Opisthorchis viverrini in the Mekong Basin. *Parasitol Int* **61**, 65-70, doi:10.1016/j.parint.2011.06.015 (2012).
72 Elkins, D. B., Haswell-Elkins, M. & Anderson, R. M. The epidemiology and control of intestinal helminths in the Pulicat Lake region of Southern India. I. Study design and pre- and post-treatment observations on Ascaris lumbricoides infection. *Trans R Soc Trop Med Hyg* **80**, 774-792 (1986).
73 Sripa, B. *et al.* Elevated plasma IL-6 associates with increased risk of advanced fibrosis and cholangiocarcinoma in individuals infected by Opisthorchis viverrini. *PLoS Negl Trop Dis* **6**, e1654, doi:10.1371/journal.pntd.0001654 (2012).
74 Linke, S., Lenz, J., Gemein, S., Exner, M. & Gebel, J. Detection of Helicobacter pylori in biofilms by real-time PCR. *Int J Hyg Environ Health* **213**, 176-182, doi:10.1016/j.ijheh.2010.03.006 (2010).
75 Yamaoka, Y. *et al.* Helicobacter pylori in North and South America before Columbus. *FEBS Lett* **517**, 180-184 (2002).

76 Thompson, J. D., Higgins, D. G. & Gibson, T. J. CLUSTAL W: improving the sensitivity of progressive multiple sequence alignment through sequence weighting, position-specific gap penalties and weight matrix choice. *Nucleic Acids Res* **22**, 4673-4680 (1994).
77 Saitou, N. & Nei, M. The neighbor-joining method: a new method for reconstructing phylogenetic trees. *Mol Biol Evol* **4**, 406-425 (1987).
78 Jones, D. T., Taylor, W. R. & Thornton, J. M. The rapid generation of mutation data matrices from protein sequences. *Comput Appl Biosci* **8**, 275-282 (1992).
79 Tamura, K., Stecher, G., Peterson, D., Filipski, A. & Kumar, S. MEGA6: Molecular Evolutionary Genetics Analysis version 6.0. *Mol Biol Evol* **30**, 2725-2729, doi:10.1093/molbev/mst197 (2013).

Tables

Table 1. Gender and age of participants and status of infection with *Opisthorchis viverrini* and species of *Helicobacter*.

**Characteristic**	**Negative for *O. viverrini***	**Positive for *O. viverrini***	***H. pylori* +ve** (n = 267)	***H. bilis* +ve** (n = 99)	***H. hepaticus* +ve** (n =18)
• **Age in years** (mean ± SD)	• 49±9·6	• 49·6±9·4	• 48·7±10·4	• 49±10·3	• 53±10
**Gender**	•	•	•	•	•
• Female	• 135/260 (51·9%)	• 153/293 (52·2%)	• 129 (48·3%)	• 48 (48%)	• 10/18 (55·6%)
• Male	• 125/260 (48·1%)	• 140/293 (47·8%)	• 138 (51·7%)	• 52 (52%)	• 8/18 (44·5%)
• *P*	• 0·38	• 0·28	• 0·44	• 0·57	• 0·51

Table 2. Prevalence of *Helicobacter* spp. and virulence factors in study participants, presented for each of five levels of intensity of infection with *Opisthorchis viverrini*.

**EPG *O. viverrini***	**0**	**1-100**	**101-500**	**501-1,000**	**>1,000**	***P* for trend**
**Level of infection intensity**	(n = 260)	(193)	(73)	(12)	(15)	
16S rRNA	125(48·1%)	135 (69·6%)	62 (84·9%)	11 (91·7%)	15 (100%)	•<br>•
OR	1	2·51	6·09	11·88	NA	•
95% CI	-	1·69-3·72	3·06-12·09	1·51-93·36	NA	0·06
P	-	< 0·001	< 0·001	0·003	< 0·001	
ureA H. pylori†	77 (29·6%)	110 (57%)	57 (78·1%)	10 (83·3%)	13 (86·7%)	< 0·001
OR	1	3·14	8·47	11·88	15·45	
95% CI	-	2·13-4·65	4·58-15·66	2·54-55·51	3·40-70·09	
P	-	< 0·001	< 0·001	< 0·001	< 0·001	
H. bilis†	14 (5·4%)	45 (23·3%)	23 (31·5%)	7 (58·3%)	10 (66·7%)	•<br>•
OR	1	5·34	2·36	24·6	35·14	•
95% CI	-	2·84-10·07	1·18-4·72	6·92-87·39	10·57-116·80	< 0·001
P	1	< 0·001	0·02	< 0·001	< 0·001	
H. hepaticus	5 (1·9%)	3 (1·6%)	5 (6·8%)	2 (16·7%)	3 (20%)	P = 0·08
OR	1	0·81	3·75	10·2	12·75	
95% CI	-	0·19-3·41	1·06-13·33	1·76-59·13	2·72-59·71	

P	-	0·53	·	0·03	0·006	
H. pylori + H. bilis†	11 (4·2%)	40 (20·7%)	23(31·5%)	6 (50%)	10 (66·7%)	< 0·001
OR	1	5·92	10·41	22·63	45·27	
95% CI	-	2·95-11·88	4·77-22·71	6·27-81·63	13·21-155·16	
P	-	< 0·001	< 0·001	< 0·001	< 0·001	
*cag*A†	13 (5·0%)	21 (10·9%)	26 (35·6%)	6 (50%)	9 (60%)	< 0·001
OR	1	2·32	10·51	19·0	28·5	
95% CI	-	1·13-4·76	5·04-21·92	15·38-67·09	8·81-92·19	
P	-	0·02	< 0·001	< 0·001	< 0·001	
*cag*E†	7 (2·7%)	13 (6·7%)	16 (21·9%)	3 (25%)	5 (33%)	< 0·001
OR	1	2·61	10·15	12·05	18·07	
95% CI	-	1·02-6·67	3·99-25·80	2·67-54·38	4·87-66·9	
P	-	0·04	< 0·001	0·007	< 0·001	
*cag*A + *cag*E†	1 (0·4%)	9 (4·7%)	12 (16·4%)	3 (25%)	5 (33%)	< 0·001
OR	1	12·7	50·95	86·33	129·5	
95% CI	-	1·59-100·86	6·5-399·37	8·16-913·23	13·81-1214·18	
P	-	0·003	< 0·001	< 0·001	< 0·001	

P = *P*-value, OR = Odds Ratio, CI = Confidence Interval. The reference group for the analysis and to estimate *P*-values and RRR is the uninfected i.e. group, EPG *O. viverrini* = 0, where the OR is 1.

Table 3. Prevalence of *Helicobacter* species and virulence genes during infection with *Opisthorchis viverrini*, and relationships with status (grade) of hepatobiliary disease as established by abdominal ultrasonography for degree of periportal echoes. Bold type letters denote significant differences.

Helicobacter	**Grade 0 +1 (n = 241)**	**Grade +2 (n = 36)**	**Grade +3 (n = 16)**
**16S rRNA *Helicobacter* species**	179 (74·3%) •	30 (83·3%)	13 (81·3%)
RRR	1	1·62	1·63
95% CI	-	0·64-4·11	0·43-6·24
P	-	0·307	0·476
ureA H. pylori	153 (90%)	28 (90·32%)	8 (100%)
RRR	1	1·03	NA
95% CI	-	0·28-3·75	NA
P	-	0·969	0·991
***cag*A**	39 (25·32%)	16 (53·33%)	6 (75%)
RRR	1	**3·38**	**9·15**
95% CI	-	1·51-7·58	1·75-47·97
P	-	**0·003**	**0·009**
***cag*E**	26 (61·9%)	7 (43·75%)	5 (83·33%)
RRR	1	0·44	3·30
95% CI	-	0·13-1·52	0·33-32·89
P	-	0·195	0·311
***cag*A + *cag*E**	21 (8·71%)	5 (13·89%)	5 (31·25%)
RRR	1	1·69	**4·9**6
95% CI	-	0·59-4·83	1·50-16·35
P	-	0·331	**0·009**

H. bilis	64 (35·75%)	14 (45·16%)	7 (53·85 %)
RRR	1	1·48	2·15
95% CI	-	0·68-3·21	0·68-6·75
P	-	0·319	0·191
H. hepaticus	12 (7·23%)	2 (7·41%)	1(9·09%)
RRR	1	1·20	1·09
95% CI	-	0·25-5·82	0·12-9·70
P	-	0·823	0·937
H. pylori + H. bilis	59 (24·38%)	14(38·89%)	6(37·50%)
RRR	1	1·95	1·96
95% CI	-	0·93-4·06	0·66-5·79
P	-	0·076	0·223

NA = not applicable, RRR = relative risk ratio, *P* = *P*-value, CI = Confidence Interval

The reference group for RRR is Grade 0+1 as grade 0 is baseline negative periductal fibrosis. Some participants were grade 1.

Table 4. Associations among genotypes of CagA of *Helicobacter pylori* and infection status with *Opisthorchis viverrini*.

Genotype	Negative for *O. viverrini* (%)	Positive for *O. viverrini* (%)
EPIYA-AB TYPE*	13/15 (86·7)	47/62 (75·8)
EPIYA-ABC TYPE	1/15 (6·7)	5/62 (8·1)
EPIYA-AB'C TYPE (B'=EPIYT)	1/15 (6·7)	10/62 (16·1)
Total	15	62

Table 5. Associations among *cag*A genotypes bearing the CagA multimerization motif (CM) and infection with *Opisthorchis viverrini*.

Genotype	Negative for *O. viverrini* (%)	Positive for *O. viverrini* (%)	Total (%)
EPIYA-AB TYPE	4/13 (30·8)	17/47 (36·2)	21/60 (35)
EPIYA-ABC	1/1 (100)	5/5 (100)	6/6 (100)
EPIYA-AB'C TYPE (B'=EPIYT) †	1/1 (100)	10/10 (100)	11/11 (100)

Table 6. *Cag*A genotypes in participants positive for liver fluke infection, and relationships with status (grade) of biliary periductal fibrosis as established by abdominal ultrasonography.

Helicobacter	**Grade 0 +1**<br>n = 242	**Grade +2**<br>n = 36	**Grade +3**<br>n = 16
***cagA* genotype**	40 (16·6%)	12 (40%)	3 (23·1%)
RRR	1	1·27*<br>**23·12****	2·31*<br>**24·36****
95% CI	-	0·21-7·61<br>2·32-230·5	0<br>1·71-347·09
P	-	0·794*<br>**0·007****	0·994*<br>**0·018****
***cagA* CM sequence**	11 (26·8%)	16 (88·9%)	6 (100%)
RRR	1	30·74	1·41
95% CI	-	5·25-180·08	0
P	-	<0·001	0·99

*, ABC type; **, AB'C type; RRR, relative risk ratio; *P* = *P*-value;
CI, confidence interval. Boldface type highlights significant differences.

Figure legends

Figure 1. Prevalence of *Helicobacter* species, *H. pylori*, *H. bilis*, *H. hepaticus* and mixed *H. pylori* and *H. bilis* in participants who were either uninfected or infected with *Opisthorchis viverrini*.

Figure 2. Phylogenetic relationship among partial CagA **sequences amplified from representative samples. Panel A.** Bootstrap consensus phylogenetic tree inferred from 500 replicates revealing four major clusters; EPIYA AB type without CagA multimerization domain (CM) (blue); EPIYA AB type containing CM domain (red); EPIYA ABC type 'Western-like' (green), and EPIYA AB'C type 'Western-like' (purple). Two Western CagA (W) and two Eastern CagA (E) reference strains detected in gastro-duodenal disease in the Thailand cohorts, and three CagA sequences isolated from bile from Thai cholangiocarcinoma (CCA) cases [42] were included (black). Branches corresponding to partitions reproduced in less than 50% bootstrap replicates were collapsed; bootstrap numbers higher than 60% are shown. Hepatobiliary disease status and *O. viverrini* infection status are shown for each sample following the indicated color code, * for egg-negative *O. viverrini* samples no ultrasound study was performed, EPG: eggs per gram of feces. **Panel B.** Multiple sequence alignment of representative partial CagA sequences belonging to four major clusters comprising the phylogram. Two representative sequences of each cluster are color-squared following the same color code as in Panel A. EPIYA domains are indicated as A, B and C, and CagA multimerization domains (CM) are highlighted (yellow).

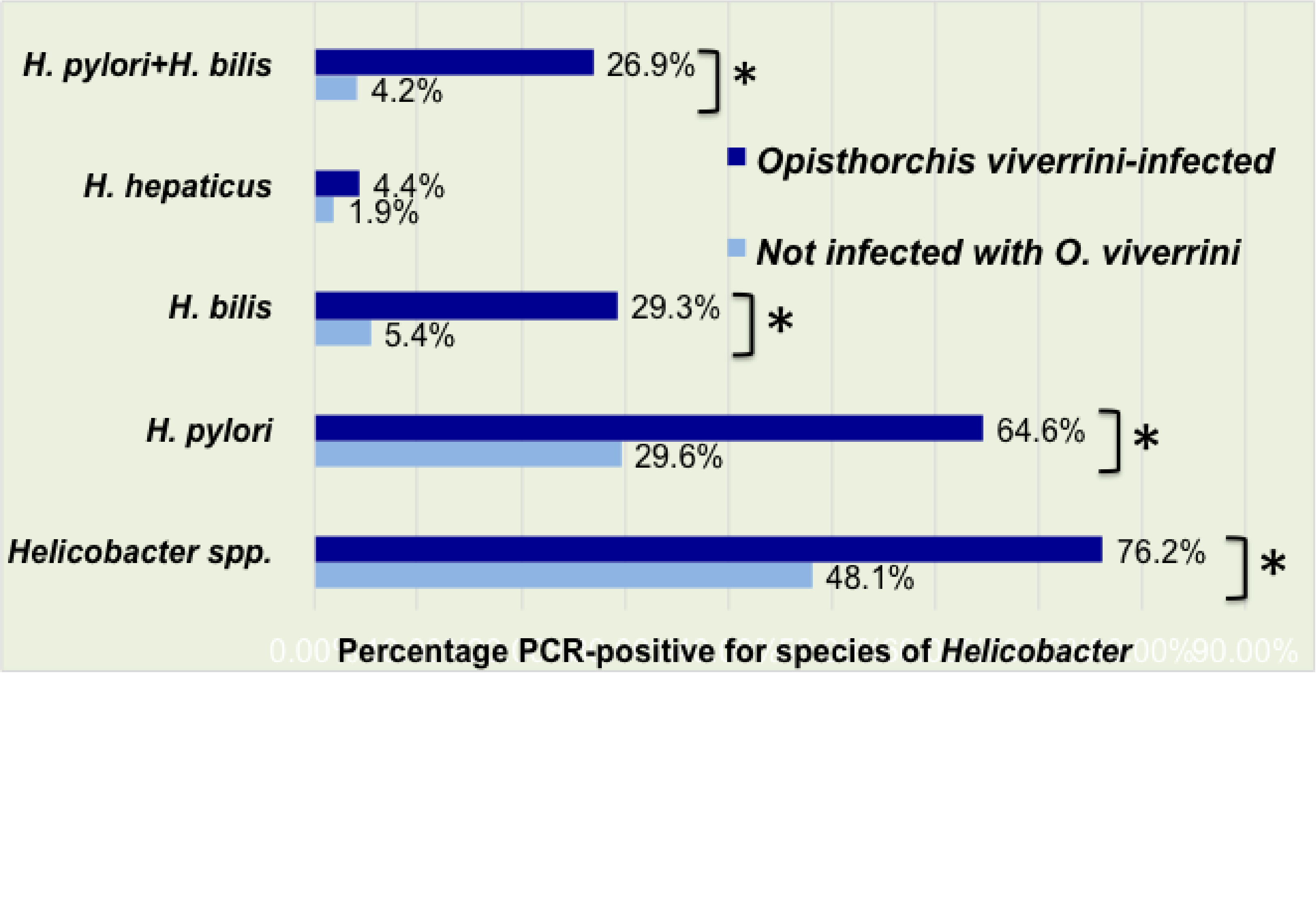

H. pylori+H. bilis
26.9%
4.2%
*
H. hepaticus
4.4%
1.9%
Opisthorchis viverrini-infected
Not infected with O. viverrini
H. bilis
29.3%
5.4%
*
H. pylori
64.6%
29.6%
*
Helicobacter spp.
76.2%
48.1%
*
90.00%
Percentage PCR-positive for species of Helicobacter

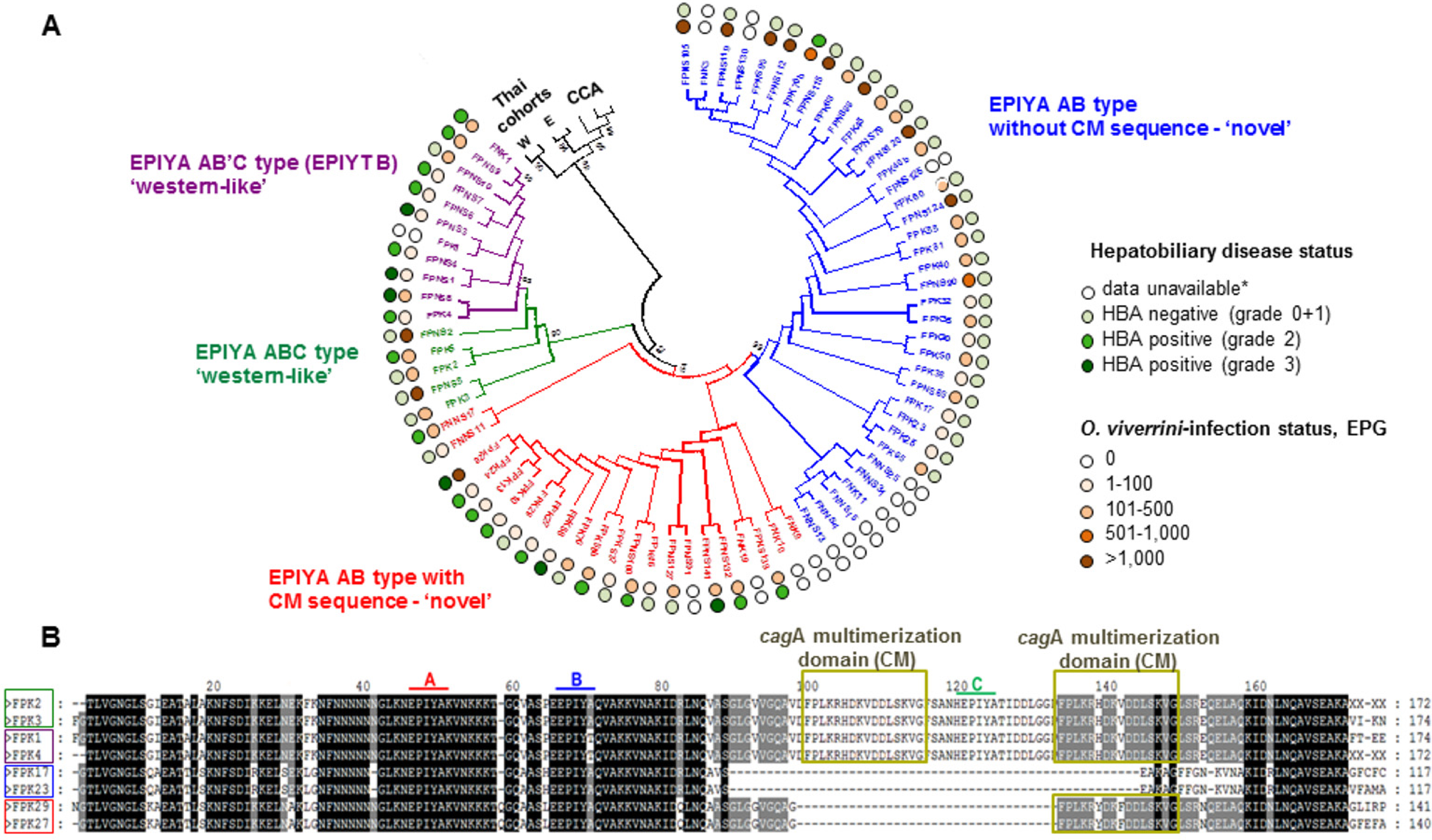
A
Thai cohorts
CCA
W
E
EPIYA AB'C type (EPIYT B)
'western-like'
EPIYA ABC type
'western-like'
EPIYA AB type
without CM sequence - 'novel'
EPIYA AB type with
CM sequence - 'novel'
Hepatobiliary disease status
data unavailable*
HBA negative (grade 0+1)
HBA positive (grade 2)
HBA positive (grade 3)
O. viverrini-infection status, EPG
0
1-100
101-500
501-1,000
>1,000
B
cagA multimerization
domain (CM)
cagA multimerization
domain (CM)
A
B
C
>FPK2
>FPK3
>FPK1
>FPK4
>FPK17
>FPK23
>FPK29
>FPK27
